# Cupriavidus pauculus Infection Associated With Extracorporeal Membrane Oxygenation in a Pediatric Patient

**DOI:** 10.7759/cureus.78203

**Published:** 2025-01-29

**Authors:** Carolina Bonilla González, Martha Álvarez-Olmos, Daniela Marulanda-Tobar, Juan P Londoño

**Affiliations:** 1 Pediatric Critical Care, Fundación Santa Fe de Bogotá, Bogotá, COL; 2 Pediatric Infectious Diseases, La Cardio, Bogotá, COL; 3 Emergency, La Cardio, Bogotá, COL; 4 Medical Education, Universidad El Bosque, Bogotá, COL

**Keywords:** bacteremia, ecmo, gram-negative bacillus, nosocomial infection, pediatric

## Abstract

We describe a critically ill neonate in the postoperative period of complex cardiovascular surgery who required extracorporeal membrane oxygenation (ECMO) and developed bacteremia caused by *Cupriavidus pauculus*. This uncommon infection in pediatric patients associated with ECMO highlights the diagnostic suspicion of bacteremia due to this non-fermentative Gram-negative bacillus. The report emphasizes early clinical evaluation and treatment and underscores the need for strict protocols to disinfect ECMO circuits, alerting health institutions to the potential transmission risk through these systems.

## Introduction

Extracorporeal membrane oxygenation (ECMO), as a life support therapy in critically ill patients, carries a high risk of complications associated with the technique and the patient’s critical condition. One of these risks is nosocomial infections, most commonly associated with coagulase-negative *Staphylococci*, followed by *Candida *spp. and *Pseudomonas aeruginosa* [1-3].

The diagnosis of infections in patients undergoing ECMO therapy can be challenging due to the lack of specific early signs and symptoms and the low specificity of acute inflammatory biomarkers.

*Cupriavidus pauculus* is an unusual microorganism in humans, typically isolated from environmental sources, especially mineral water and soil. However, it has been identified as a potential nosocomial pathogen associated with ECMO circuit contamination [1-3].

This case report describes a newborn with bacteremia caused by a Gram-negative bacillus that tested negative on the sepsis PCR identification panel (FilmArray® BCID 1.0, Biofire Diagnostics, USA/bioMérieux, France) but was identified as *C. pauculus* using the Vitek-2 system (bioMérieux).

To our knowledge, this is the first description of *C. pauculus* bacteremia in Latin America. This case underscores the importance of reporting such infections to improve diagnostic suspicion and provide evidence in ECMO therapy.

## Case presentation

A 48-hour-old neonate was referred to the Pediatric Cardiovascular Intensive Care Unit (PCICU) at Fundación Cardioinfantil, Bogotá, Colombia, with a diagnosis of dextro-transposition of the great arteries, restrictive foramen ovale, pulmonary hypertension, and right atrial dilatation with acceptable biventricular function. An immediate percutaneous atrioseptostomy was performed, enabling the infant to undergo another surgical procedure at eight days of life, weighing 2.8 kg. An arterial switch operation, ductus arteriosus closure, and atrial septal defect repair were performed using bypass and clamp times of 306 and 204 minutes, respectively. Postoperatively, the patient experienced a massive alveolar hemorrhage requiring right upper lobe repair and multiple blood transfusions.

Subsequently, the patient developed cardiogenic and hypovolemic shock, leading to pulseless electrical activity for 10 minutes and requiring venous-arterial ECMO therapy. Cefazolin (70 mg/8 hours) was administered as antibiotic prophylaxis before surgery. Within the first 48 postoperative hours, the patient developed significant leukopenia and lymphopenia (day 1), prompting the initiation of prophylactic trimethoprim/sulfamethoxazole (15 mg/12 hours on Monday, Wednesday, and Friday). Blood cultures, including post-membrane samples from the ECMO circuit, were positive for Gram-negative bacilli at 22 hours, and empirical cefepime (140 mg/8 hours) was initiated. A second set of blood cultures was also positive for Gram-negative bacilli in the ECMO post-membrane sample at 20 hours. The FilmArray sepsis panel (FilmArray® BCID 1.0) reported a negative result within one hour. After 48 hours of cefepime treatment, a third blood culture revealed a Gram-negative bacillus in an aerobic blood culture from the left jugular catheter. Despite negative FilmArray results and the inability of automated microbiological systems and biochemical tests to identify the microorganisms in these three blood cultures, there was a strong suspicion of bacteremia caused by a non-fermenting Gram-negative bacillus not detected by FilmArray. This led to the re-initiation of trimethoprim/sulfamethoxazole (15 mg/12 hours) alongside meropenem (85 mg/8 hours).

The microbiology laboratory subsequently identified the Gram-negative bacillus as *C. pauculus* using the Vitek 2 Compact® system (bioMérieux). This microorganism had not been previously isolated at our institution. A manual antibiogram revealed possible sensitivity to ceftazidime, piperacillin/tazobactam, imipenem, and ciprofloxacin, with resistance to ampicillin and meropenem, compared to Clinical and Laboratory Standards Institute (CLSI) cut-off points for *P. aeruginosa* and related organisms.

Treatment with piperacillin/tazobactam (280 mg/6 hours) was initiated, while trimethoprim/sulfamethoxazole was continued. The patient developed significant multilobar consolidation, predominantly in the right apical lobe, attributed to mucous plugs and blood clots associated with alveolar hemorrhage. A fibrobronchoscopy was performed without complications. After 10 days, ECMO therapy was discontinued. Follow-up blood cultures were obtained 72 hours after antibiotic initiation, and no Gram-negative bacilli or other pathogens were isolated from blood, urine, orotracheal secretions, or peritoneal fluid cultures.

Water samples from the reservoir and thermoregulator were analyzed by an industrial laboratory, but no microorganisms were isolated. Unfortunately, logistical constraints prevented culturing the oxygenation membrane.

After ECMO withdrawal, the patient developed cardiogenic shock, multifactorial respiratory failure, and severe oxygenation impairment. High-frequency ventilation, nitric oxide, and renal replacement therapy (peritoneal dialysis) were required. The patient subsequently developed refractory hypoxemia progressing to multiorgan failure, leading to death. No additional isolates of *C. pauculus* were obtained from subsequent cultures, nor were other cases of infection caused by this microorganism reported.

A multidisciplinary infection control approach was initiated, including retraining on patient care procedures, revision of disinfection protocols, and reevaluation of thermoregulator use.

Table 1 presents the results of complete blood counts, blood cultures, and the implemented antibiotic interventions, providing an overview of the management of *C. pauculus* infection in this case.

**Table 1:** Complete blood count, blood cultures, and antibiotic interventions in the management of Cupriavidus pauculus infection in a pediatric extracorporeal membrane oxygenation (ECMO) patient ECMO: extracorporeal membrane oxygenation; TMP/SMX: trimethoprim/sulfamethoxazole

Day	Event	Complete blood count leukocytes (10³/µL) normal range: 5.0-21.0 × 10³/µL	Complete blood count neutrophils (10³/µL) normal range: 1.5-10.0 × 10³/µL	Complete blood count lymphocytes (10³/µL) normal range: 2.0-17.0 × 10³/µL	Procalcitonin (PCT) (ng/mL) <0.5 ng/mL: Low risk of infection >2 ng/mL: High risk of infection	Blood cultures aerobic	Blood cultures anaerobic	Sepsis FilmArray	Antibiotics
	Pre-ECMO	14.900	7.360	4.300					
	Surgery	2.250	1.550	400					Prophylactic cefazolin
1	ECMO	1.730	960	360	0.67				
3	ECMO	5.410	4.250	510		Post-membrane ECMO: *Cupriavidus pauculus* at 22 hours	Negative		Cefepime + prophylactic TMP/SMX
4	ECMO	7.300	5.640	620	1.5	Post-membrane ECMO: *Cupriavidus pauculus* at 20 hours	Negative	No detections	Cefepime + prophylactic TMP/SMX
6	ECMO	7.350	4.940	560	1.08	Jugular catheter: *Cupriavidus pauculus* at 27 hours	Negative		Cefepime + prophylactic TMP/SMX
8	ECMO	7.930	6.140	440	1.94				Cefepime + prophylactic TMP/SMX
10	ECMO	10.700	8.830	220		Post-membrane ECMO: *Cupriavidus pauculus* at 21 hours	Negative	No detections	Piperacillin tazobactam + therapeutic TMP/SMX
12	Post-ECMO	13.800	10.100	840	1.83				Piperacillin tazobactam + therapeutic TMP/SMX
15	Post-ECMO	18.400	14.900	910		Femoral line: *Staphylococcus epidermidis* at 24 hours	Jugular catheter: *Staphylococcus epidermidis* at 29 hours		Piperacillin tazobactam + therapeutic TMP/SMX + Linezolid
17	Post-ECMO	18.400	16.000	530	4.37				Piperacillin tazobactam + therapeutic TMP/SMX + Linezolid
18	Post-ECMO	18.200	16.800	410		Femoral line: *Staphylococcus epidermidis* at 45 hours	Jugular catheter: *Staphylococcus epidermidis* at 48 hours		Piperacillin tazobactam + therapeutic TMP/SMX + Linezolid + Caspofungin
20	Post-ECMO	11.700	10.100	450		Femoral line: *Staphylococcus epidermidis* at 41 hours			Piperacillin tazobactam + therapeutic TMP/SMX + Linezolid + Caspofungin

## Discussion

ECMO is a life-support method for reversible cardiac and pulmonary failure in neonates, children, and adults. However, it is associated with significant risks, including infections. Despite advancements in disinfection protocols and infection control, these risks persist [4].

Healthcare-associated infections (HAIs), including bloodstream infections, ventilator-associated pneumonia, and cannulation site infections, are among the most frequent complications of ECMO therapy, surpassing hemorrhagic or mechanical complications. The incidence of these infections ranges from 10.1 to 116.2 per 1,000 ECMO days [4].

The diagnosis of infections in ECMO patients is complicated due to the thermal control characteristics of the system, which can mask fever as a clinical sign. Additionally, immune system activation caused by blood exposure to the artificial ECMO surfaces can elevate inflammatory biomarkers, complicating the differentiation between infection and systemic inflammation. Although routine blood cultures have been proposed as a preventive measure, the Extracorporeal Life Support Organization (ELSO) does not recommend this practice due to insufficient evidence of benefits and associated costs. Instead, blood cultures should only be performed when there is clinical suspicion of infection.

In 2011, ELSO published data from over 100 centers and 20,741 patients of all ages, reporting infection rates of 10.1 per 1,000 ECMO days in neonates, 20.8 per 1,000 ECMO days in pediatric patients, and 30.6 per 1,000 ECMO days in adults [5]. Specifically, venoarterial ECMO is associated with higher infection rates compared to other ECMO modes [1].

In all populations, the most frequently isolated pathogens include coagulase-negative staphylococci (15.9%), Candida species (12.7%), *P. aeruginosa* (10.5%), *Staphylococcus aureus *(9.4%), *Enterobacter *spp. (5.7%), *Klebsiella *spp. (4%), *Enterococcus *spp. (4%), and* Escherichia coli *(3.9%) [1-3].

In neonatal populations, nosocomial infections related to ECMO are associated with prolonged hospital stays [6] and higher mortality rates, with rates varying between 35% and 52% in infected patients compared to 13% and 21% in non-infected patients [4]. Moreover, the infecting microorganism significantly influences outcomes, with gram-negative bacteria and fungi associated with lower survival rates [7-11].

This report presents the case of an eight-day-old neonate who required ECMO support following arterial switch surgery, patent ductus arteriosus closure, and atrial septal defect closure. Blood cultures isolated *C. pauculus*, a rare, gram-negative, aerobic, non-fermentative, non-spore-forming bacillus. This microorganism is typically found in environmental sources such as soil and mineral water and is an unusual pathogen in humans. *C. pauculus* is considered opportunistic and primarily affects immunocompromised individuals or those undergoing invasive procedures and broad-spectrum antibiotic treatment [12,13].

*C. pauculus* was first described in 1987 as *Cupriavidus necator*. Subsequent taxonomic revisions based on phenotypic and genotypic analyses reclassified it under various genera, including Ralstonia and Wautersia. Current phylogenetic data support its classification within the Cupriavidus genus, with the species name pauculus, as per the International Code of Nomenclature of Bacteria [14].

The antibiotic susceptibility data for *C. pauculus *indicate sensitivity to broad-spectrum beta-lactams, trimethoprim/sulfamethoxazole, and quinolones, while resistance has been observed to aminoglycosides, ampicillin, and aztreonam. Variable susceptibility to macrolides, tetracyclines, and chloramphenicol has also been reported. In this case, the susceptibility patterns were consistent with previously reported data [15]. Worldwide, in the literature review from 1985 to 2024, only five cases associated with ECMO support have been reported [16,17], four of which originated from the same center. In contrast, to the best of our knowledge, 12 pediatric cases of *C. pauculus* infection unrelated to ECMO have been described, the majority occurring in patients with predisposing risk factors and immunocompromised conditions [12,18-21].

## Conclusions

This case report highlights the potential for nosocomial pathogen transmission through the ECMO circuit and underscores the importance of early suspicion and accurate microorganism identification in infections acquired during this support. Although we were unable to isolate *C. pauculus* from the ECMO equipment, clinical and microbiological evidence suggests a possible association with extracorporeal support, similar to other reports documenting contamination of the thermoregulatory reservoir as a source of infection. Furthermore, this case emphasizes the need for active surveillance of contamination sources to optimize disinfection protocols and implement infection control strategies. The implementation of such measures is crucial to reducing the risk of infections in critically ill patients on ECMO and mitigating their impact on morbidity and mortality.

Early detection and the use of rapid identification tests are essential to optimizing clinical responses, ensuring efficient resource utilization, and guiding appropriate antimicrobial therapy. Additionally, a multidisciplinary approach to infection control, complemented by active microbiological surveillance and the implementation of tailored preventive measures, is critical in limiting the spread of rare pathogens in intensive care units and among patients receiving extracorporeal support.
